# Multimodal nonlinear correlates of behavioural symptoms in frontotemporal dementia

**DOI:** 10.1007/s11682-024-00913-7

**Published:** 2024-09-07

**Authors:** Giovanna Zamboni, Irene Mattioli, Zobair Arya, Manuela Tondelli, Giulia Vinceti, Annalisa Chiari, Mark Jenkinson, Edward D. Huey, Jordan Grafman

**Affiliations:** 1 Università di Modena e Reggio Emilia, Modena, Italy; 2 Azienda Ospedaliero Universitaria di Modena, Modena, Italy; 3 Nuffield Department of Clinical Neurosciences, University of Oxford, Oxford, UK; 4 Departments of Psychiatry and Human Behavior, Alpert Medical School of Brown University, Providence, USA; 5 Shirley Ryan AbilityLab & Northwestern University Feinberg School of Medicine, Chicago, IL USA; 6 Department of Biomedical, Metabolic and Neural Sciences, University of Modena and Reggio Emilia, Via Giardini 1355, Modena, 41126 Italy

**Keywords:** Frontotemporal dementia, Behavioral and psychological symptoms of dementia, Behavior, Fusion, Multimodality, PET, MRI

## Abstract

Studies exploring the brain correlates of behavioral symptoms in the frontotemporal dementia spectrum (FTD) have mainly searched for linear correlations with single modality neuroimaging data, either structural magnetic resonance imaging (MRI) or fluoro-deoxy-D-glucose positron emission tomography (FDG-PET). We aimed at studying the two imaging modalities in combination to identify nonlinear co-occurring patterns of atrophy and hypometabolism related to behavioral symptoms. We analyzed data from 93 FTD patients who underwent T1-weighted MRI, FDG-PET imaging, and neuropsychological assessment including the Neuropsychiatric Inventory, Frontal Systems Behavior Scale, and Neurobehavioral Rating Scale. We used a data-driven approach to identify the principal components underlying behavioral variability, then related the identified components to brain variability using a newly developed method fusing maps of grey matter volume and FDG metabolism. A component representing apathy, executive dysfunction, and emotional withdrawal was associated with atrophy in bilateral anterior insula and putamen, and with hypometabolism in the right prefrontal cortex. Another component representing the disinhibition versus depression/mutism continuum was associated with atrophy in the right striatum and ventromedial prefrontal cortex for disinhibition, and hypometabolism in the left fronto-opercular region and sensorimotor cortices for depression/mutism. A component representing psychosis was associated with hypometabolism in the prefrontal cortex and hypermetabolism in auditory and visual cortices. Behavioral symptoms in FTD are associated with atrophy and altered metabolism of specific brain regions, especially located in the frontal lobes, in a hierarchical way: apathy and disinhibition are mostly associated with grey matter atrophy, whereas psychotic symptoms are mostly associated with hyper-/hypo-metabolism.

**Supplementary Information:**

The online version contains supplementary material available at 10.1007/s11682-024-00913-7.

## Introduction

The constellation of behavioral symptoms occurring in the syndromes of the FTD spectrum is extremely variable, and individual patients may present some behavioural symptoms without ever presenting others (Barker et al., 2022; Rascovsky et al., 2011). Studies have demonstrated clinical-anatomical correspondences by relating changes in a single neuroimaging modality with questionnaires measuring the severity of single behavioural symptoms in a linear way (Levenson et al., 2014; Rosen et al., 2005; Whitwell et al., 2007; Zamboni, 2016). Clinical experience, however, suggests that behavioural symptoms tend to cooccur in variable combinations across patients. In addition, the association between the severity of the symptoms and the brain may not be linear. The majority of previous studies on behavioural symptoms in the FTD spectrum have focussed on structural magnetic resonance imaging (MRI) with the assumption that behavioural variability can be fully explained by grey matter atrophy, a marker of neurodegeneration (Lansdall et al., 2017; Rosen et al., 2005; Zamboni et al., 2008). In parallel, other studies have independently tried to link behavioural symptoms to regional hypometabolism measured with (^18^F)-2-fluoro-deoxy-D-glucose positron emission tomography (FDG-PET), a marker of early synaptic dysfunction (Cerami et al., 2015; Ruby et al., 2007). The lines of research on the correlates of behavioural symptoms in FTD based on the two different imaging modalities (MRI and FDG-PET) have progressed independently.

In the present study we explored whether the variability of behavioural symptoms in FTD is better captured by changes in brain atrophy measured with structural MRI or brain hypometabolism measured with FDG-PET. We first identified modes of variation (components) explaining the variability of behavioural symptoms in patients with FTD using several different behavioural questionnaires. We then studied how the identified components relate to changes in both brain structure (MRI) and metabolism (FDG-PET), using a novel multimodal decomposition technique (Arya, 2019; Arya et al., 2019) that allowed us to also identify nonlinear relationships.

## Materials and methods

### Subjects

We enrolled patients seen at the Cognitive Neuroscience Section of the National Institute of Neurological Disorders and Stroke, NIH, between 2002 and 2009. In order to be included they needed to have a diagnosis of FTD according to the criteria available at the time (Neary et al., 1998) but also according to subsequent criteria for FTD spectrum disorders, which include PPA and behavioural variant of FTD (Gorno-Tempini et al., 2011; Rascovsky et al., 2011). During a single 1-week visit at the NIH, patients underwent brain MRI and FDG-PET scanning, and extensive neuropsychological evaluation including Neuropsychiatric Inventory (NPI) (Cummings et al., 1994), Frontal Systems Behavior Scale (FrSBe) (Grace & Malloy, 2001), and Neurobehavioral Rating Scale (NBRS) (Levin et al., 1987). All consecutive subjects with both MRI and FDG-PET data and behavioural assessment were included.

### Statistical analysis

Behavioural data were analysed using IBM SPSS Statistics 23 for Mac. We fed all the neurobehavioral data (including the 3 scores from the FrSBe, the 27 items of the NBRS, the 10 items of the NPI) by means of a Principal Component Analysis (PCA), with direct oblimin rotation (i.e., a nonorthogonal rotation method which allows the extracted neurobehavioral components to be correlated). Statistical significance was set at *p* < 0.05.

### Imaging acquisition and preprocessing

A 1.5-tesla GE MRI scanner (GE Medical Systems, Milwaukee, WI) and standard quadrature head coil were used to obtain MRI images. A T1-weighted spoiled gradient echo sequence was used to generate 124 contiguous 1.5-mm-thick axial slices (repetition time = 6.1 msec; flip angle = 20°; field of view = 240 mm; matrix size = 256 × 256 × 124). MRI data were analysed with FSL-VBM.

A GE Advance three-dimensional PET scanner was used to acquire FDG-PET images (4.25-mm slice separation, 35 slices, axial field of view 15.3 cm, transverse field of view 55.0 cm). Subjects were made to fast from midnight before the scan and had no caffeine, alcohol, or nicotine for 24 h before the scan. The subject was given an intravenous injection of 5 millicuries of FDG. Starting with the time of injection and continuing through the cerebral uptake period and subsequent scan, 25 arterial blood specimens were taken at fixed intervals for assay of plasma radioactivity and glucose content. FDG-PET data were brain extracted, registered to the same subject’s T1 MRI, then to the study grey matter template obtained from FSL-VBM. Images were intensity normalised using a cerebellum mask. For all analyses VBM and PET unilinear analyses, we accepted a threshold of *p* < 0.05 Threshold-Free Cluster Enhancement (TFCE) correction.

### Unimodal imaging analysis

Unimodal Voxel-based Morphometry (VBM) *linear* analyses were used to identify regions of significant *linear* correlation between grey matter density and each of the three components obtained from the PCA, by applying permutation-based non-parametric inference (Nichols & Holmes, 2002). The model included age, sex, and the Mattis-DRS total score (as a measure of global dementia severity) as covariates of no interest. Unimodal PET *linear* analyses were used to identify regions of significant linear correlation between regional metabolism and the three components, with the same covariates of no interest. In addition, PET unimodal analyses were also controlled for local atrophy by including in the model the grey matter images from the VBM as an additional voxelwise covariate of no interest.

### Multimodal imaging analysis

Multimodal VBM and PET *non-linear* analyses were performed with a new technique aimed at identifying voxel “trajectories”, i.e. the rates of change of the voxel values with respect to a considered variable, to reveal a set of spatial maps where the voxels indicate locations that change in the same way across each modality (Arya, 2019; Arya et al., 2019). This multimodal decomposition technique is explicitly informed by a single variable of interest at a time (in our case the behavioural components obtained from the PCA) and is then applied to MRI and FDG-PET images. This technique assumes that the trajectory of a given voxel’s values, as a function of the variable of interest, is mainly governed by one component, out of a possible number of trajectories. The aim is to estimate the spatial maps and trajectories/subject weights associated with these number of trajectories. A step-by-step description of this multimodal decomposition technique is provided in the Supplementary material and is graphically represented in Supplementary Fig. 1.

Existing decomposition techniques will typically produce trajectories that have no specific relationship to the variable of interest and any associations with such a variable must be established by post-hoc manipulations that are often suboptimal, indirect and not based on an underlying model. This fusion method is based on clustering methodology that only returns trajectories that are directly optimised to be related to the variable of interest and shows explicit connections across modalities. The method had been previously validated with both simulated data and real data (the UK biobank) with respect to ageing as variable of interest (Arya, 2019; Arya et al., 2019). When applied to simulated data, it was able to accurately estimate trajectories and associated spatial maps. When applied to real data (the UK biobank dataset), the method was able to estimate trajectories and associated regions that were in agreement with previously published results and were a lot easier to interpret (Arya, 2019).

## Results

Ninety-three patients (48 men [51.6%], 45 women [48.4%]; 89 Caucasian, 4 non-Caucasian; 91 non-Hispanic, 2 Hispanic; Table 1) were included in the study. Sixty-seven patients were clinically characterized as having bvFTD (72.0%), five presented with both bvFTD and motor neuron disease (5.4%), seventeen were characterized as the non-fluent variant of PPA (5.4%), and four with the semantic variant of PPA (4.3%).

**Table 1:** Clinical and behavioural characteristics

	*N*	Mean	Std. Dev.	Minimum	Maximum
Age at assessment	93	59.7	8.5	41.0	85.0
Education, years	93	15.8	2.8	10.0	20.0
Age at symptoms onset	93	55.3	8.5	39.0	83.0
Duration of disease, months	92	5.0	5.2	1.0	45.0
Mattis-DRS	88	101.6	28.2	11.0	143.0
FrSBe Total	81	138.0	37.8	42.0	200.0
Apathy		14.0	46.9	12.6	69.0
Disinhibition		15.0	35.6	10.8	63.0
Executive Dysfunction		17.0	59.4	15.1	83.0
NBRS_Total Pathology Score	89	57.5	14.2	30.0	98.0
NPI_Total Score	87	28.7	16.6	0.0	72.0

DRS, Dementia Rating Scale; FrSBe, Frontal Systems Behavior Scale; NBRS, Neurobehavioral Rating Scale; NPI, Neuropsychiatric Inventory

### Behavioural results

The PCA with direct oblimin rotation was computed on the scores of the neurobehavioral tests (NPI, FrSBe, NBRS) and gave good indicators of factorability (Kaiser-Meyer-Olkin Measure of Sampling Adequacy: 0.56, Bartlett’s Test of Sphericity: Approx. Chi Square 1907.707, df 780, *p* < 0.001), and the residuals indicate that the solution was a good one. Three components with an eigenvalue of greater than 3.0 were found (Supplementary Fig. 2 and Supplementary Table 1). The following three components were identified:

- Component 1 loaded ‘decreased motivation’, ‘emotional withdrawal’, and ‘blunted affect’ of the NBRS, and ‘apathy’ of the FrSBe and NPI. It also loaded ‘inattention’, ‘conceptual disorganisation’ and ‘poor planning’ of the NBRS, and ‘executive dysfunction’ of the FrSBe. We labelled it as *Apathy*.
- Component 2 loaded, on its negative end, ‘disinhibition’ from the FrSBe and NPI, and on its positive end, ‘expressive deficit’, ‘speech articulation defect’ and ‘depressive mood’ of the NBRS. We labelled it as *Disinhibition versus depression/mutism*.
- The last component loaded ‘hallucinations’ and ‘delusions’ of the NPI, as well as ‘hallucinatory behaviour’, ‘unusual thought content’, and ‘suspiciousness’ of the NBRS. We labelled it as *Psychosis*.

Sensitivity analyses performed on the bvFTD sample only (i.e., by removing the 20 patients with PPA) yielded overlapping results.

### Unimodal imaging results

**Fig. 1:**
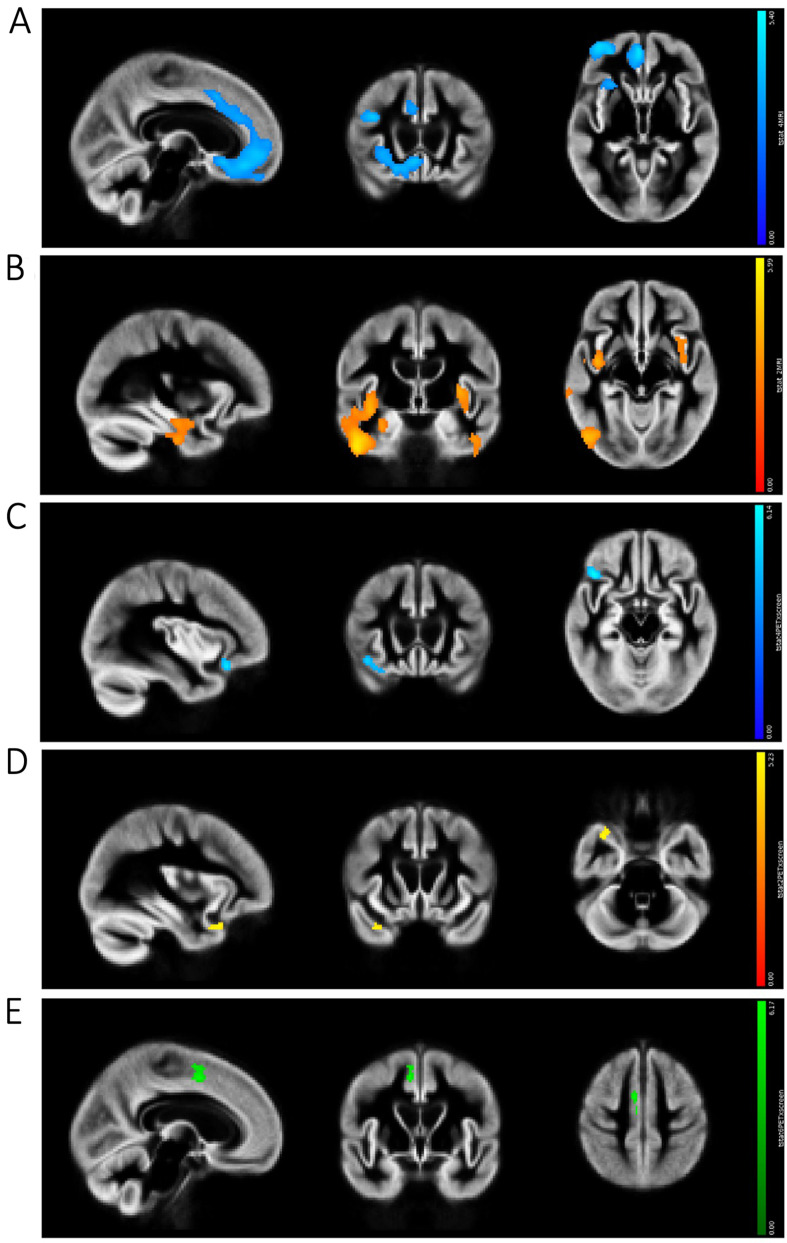
Unimodal imaging results. **(A)** Voxel based morphometry (VBM) results for Component 1 (in blue), showing atrophy associated with *Apathy*; **(B)** VBM results for Component 2 (in red-yellow), showing atrophy associated with *Disinhibition versus depression/mutism*; **(C)** PET results for Component 1 (in blue), showing regions of reduced metabolism associated with *Apathy*; **(D)** PET results for Component 2 (in yellow), showing regions of reduced brain metabolism associated with disinhibition (i.e., negative values from Component *Disinhibition versus depression/mutism were associated with reduced metabolism)*; **(E)** PET results for Component 3 (in green), showing regions of reduced brain metabolism associated with *Psychosis*

Preliminary VBM linear analyses showed that Component 1 (*Apathy*) was associated with atrophy in the medial prefrontal cortex (cingulate and orbitofrontal cortices) especially on the right side, and right posterior middle frontal gyrus (Fig. 1A). Component 2 (*Disinhibition versus depression/mutism*) was associated with atrophy in the posterior insula bilaterally and middle-inferior temporal gyrus, especially in the right hemisphere on the end of increasing disinhibition and euphoria (Fig. 1B), whereas the opposite end did not lead to significant results. Component 3 (*Psychosis*) was not associated with significant unilinear atrophy.

PET correlational analyses showed that increasing values on Component 1 were associated with hypometabolism in two discrete regions in the right anterior cingulate cortex and right lateral orbitofrontal cortex (Fig. 1C). Decreasing values on Component 2, indicating greater disinhibition, were associated with hypometabolism in the right anterior temporal pole (Fig. 1D). Increasing values on Component 3 were associated with hypometabolism in the right medial posterior frontal cortex (Fig. 1E).

### Multimodal imaging results

**Fig. 2:**
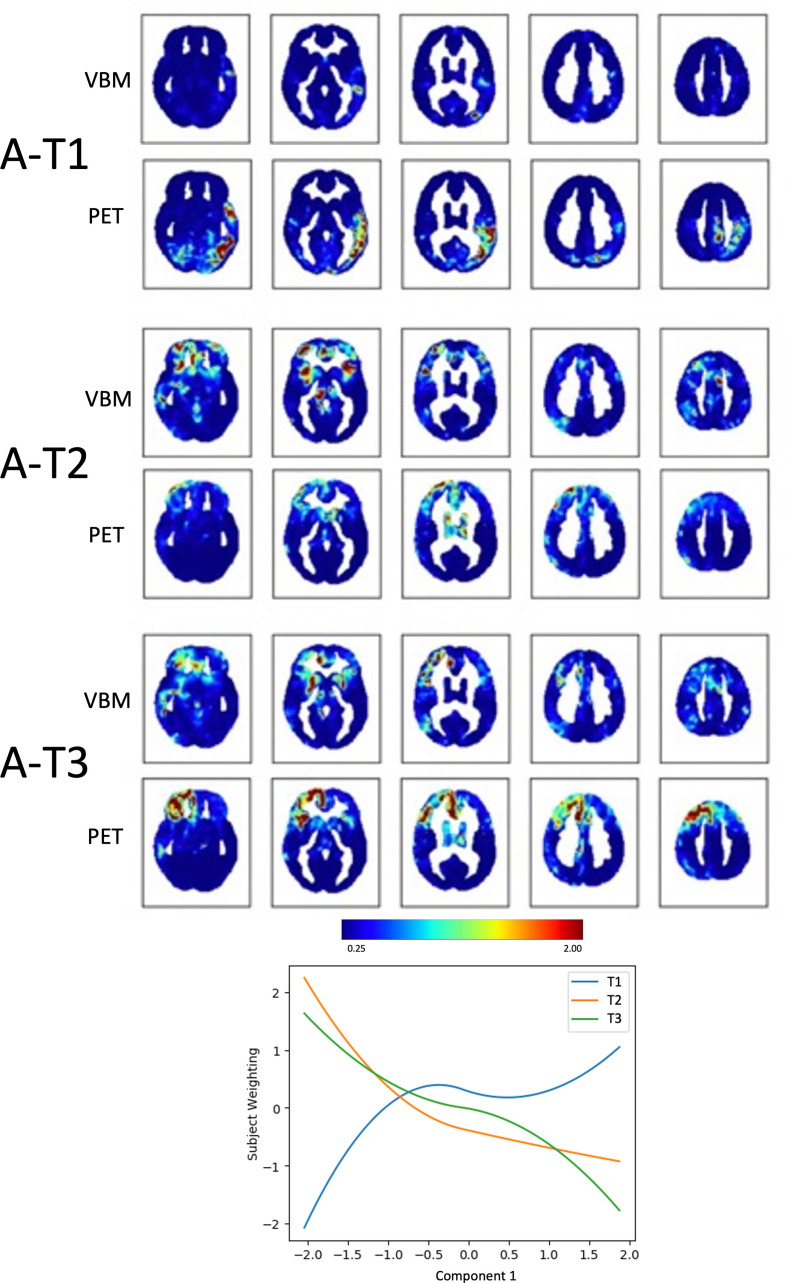
Fusion analysis on *Apathy ****(A)***Spatial maps of clusters of voxels changing in tandem across the two modalities (VBM and PET) along the three trajectories (T) that “fit best” with the variability along Component 1, *Apathy (A)*. The plot shows how the identified trajectories relate to Component 1. The color bar represents the likeliness for that voxel to belong to a given trajectory in terms of inverse and normalised squared Euclidean distance to each of the estimated centroid trajectories (varying from negligible [0 mm, blue], to average [0.6–0.8 mm, green-yellow], to high [> 1.2 mm, red])

**Fig. 3:**
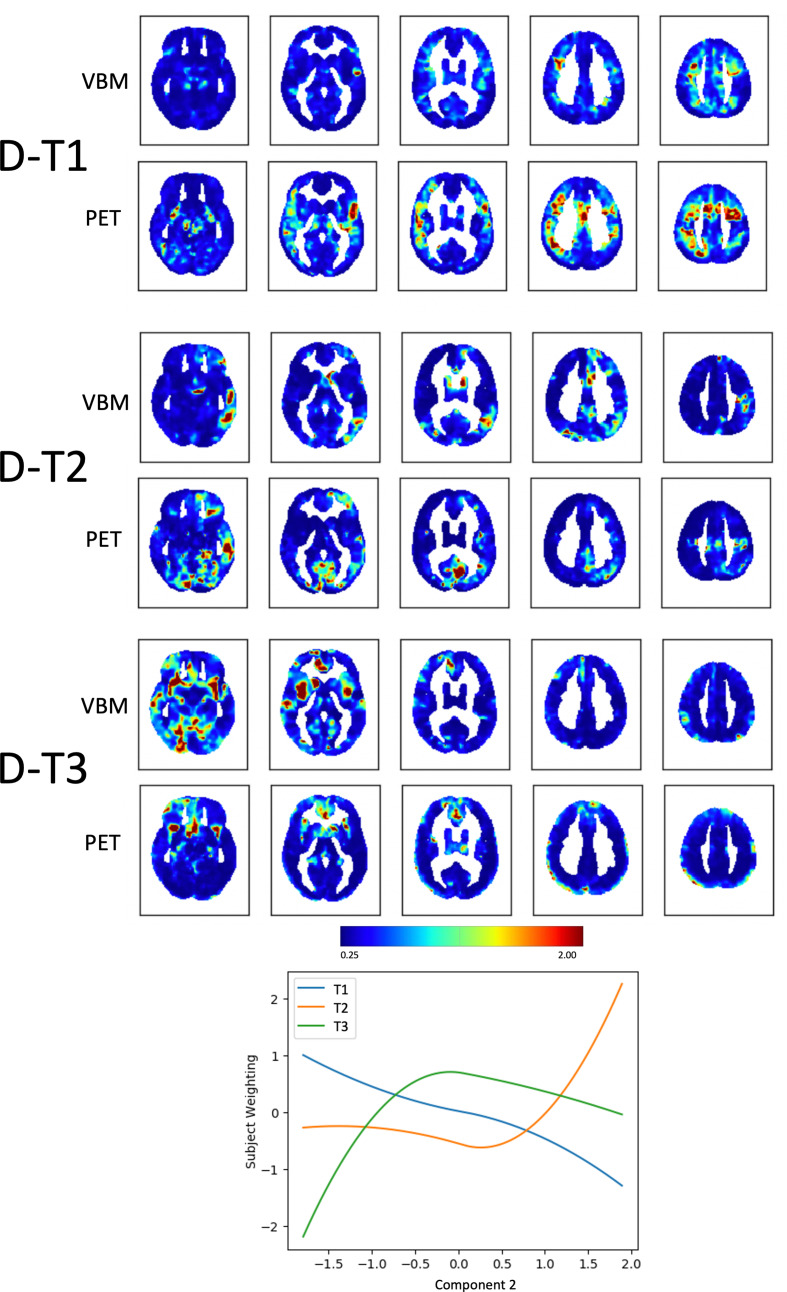
Fusion analysis on *Disinhibition versus depression/mutism (D) *Spatial maps of clusters of voxels changing in tandem across the two modalities (VBM and PET) along the three trajectories (T) that “fit best” with the variability along Component 2, *Disinhibition versus depression/mutism (D)*. The plot shows how the identified trajectories relate to Component 2. The color bar represents the likeliness for that voxel to belong to a given trajectory in terms of inverse and normalised squared Euclidean distance to each of the estimated centroid trajectories (varying from negligible [0 mm, blue], to average [0.6–0.8 mm, green-yellow], to high [> 1.2 mm, red])

Component 1, *Apathy (A)*, could be explained by 3 multimodal trajectories, representing clusters of voxels changing in the same way across the two modalities, VBM and PET, in relation to the behavioural nuances captured by the component (see the dendrogram obtained with hierarchical clustering in Supplementary Fig. 3A). Subject weights were plotted against Component 1 to visualize the extracted trajectories (Fig. 2; Table 2). The multimodal results were visualized using spatial maps both for VBM and PET of the various trajectories. In these maps the highlighted regions represent clusters of voxels that “fit best” with the variability along Component 1, *Apathy*. Two trajectories (A-T2 and A-T3) captured clusters of voxels in which volume and metabolism decrease with increasing values of *Apathy*: these voxels were mainly located in the right prefrontal cortex for both modalities. More precisely, A-T2 showed that the higher the *Apathy* component value, the lower the grey matter volume in the anterior insula bilaterally and in the right anterior cingulate cortex (from VBM), paired to decreased metabolism in bilateral frontal poles and right thalamus (from PET). A-T3 showed that the higher the *Apathy* component value, the lower the volume in the right cingulate and right putamen, paired to decreased metabolism in the whole right prefrontal cortex (Fig. 3). The first trajectory (A-T1) was associated mainly with PET and captured voxels in which metabolism *increased* with increasing values of *Apathy*: these voxels were located in the left temporal and temporo-parietal regions.

**Table 2:** Summary of the multimodal imaging results

		VBM	PET
***Component 1*** ***Apathy***	A-T1	**right and left parahippocampal** gyrus, **left Heschl gyrus and superior temporal gyrus**, left occipital pole, left pre and post central gyri, **left precuneus**	**left inferior**, middle and superior (anterior division) **temporal gyrus**,** left supramarginal gyrus**,** left parietal operculum and left parietal lobule**, **temporal-occipital fusiform cortex**, **left lingual gyrus**, lateral **occipital cortex**, left post central e precentral
	A-T2	**right frontal orbital**, bilateral subcallosal cortex, **right and left frontal pole**, right and **left insular cortex**, left frontal operculum, **left middle frontal gyrus**, right and left superior frontal gyrus, right paracingulate cortex, **right angular gyrus**,** right hippocampus and amygdala**,** right thalamus**	right and **left frontal pole**,** right** and left **caudate** and putamen, **right thalamus**, right hippocampus
	A-T3	**right frontal medial cortex/paracingulate gyrus and cingulate**, **right putamen**, **right and left angular gyrus**, right inferior frontal gyrus, **right and left middle frontal gyrus**, right superior frontal gyrus, **right** and left **subcallosal cortex**, **right middle temporal gyrus**, right precuneus	**right orbitofrontal**,** right frontal pole**, **bilateral insular cortices**, left inferior frontal gyrus, **right** and left **cingulate gyrus**, **right** and left **superior frontal gyrus**, left middle frontal gyrus
***Component 2*** ***Disinhibition versus depression/mutism***	D-T1	**left thalamus (pulvinar)**, left calcarine cortex, **left frontal pole**, left middle and inferior frontal gyrus, **left** and right **superior frontal gyrus**, left precentral gyrus, **right cingulate gyrus (posterior division)**	**bilateral putamen**, **right thalamus**, **right caudate**, **bilateral precentral gyrus**, right supramarginal gyrus, **bilateral cingulate posterior division**, right and **left superior** and inferior **frontal gyrus**
	D-T2	left and **right inferior temporal gyrus**, left middle temporal gyrus, **left lateral occipital cortex**,** left angular gyrus**, **left** and right **cingulate gyrus**	left temporal pole, **left** and right **lingual gyrus**, left precuneus, left cingulate gyrus
	D-T3	right temporal fusiform cortex, right parahippocampal, **left temporal pole**, **bilateral insular cortex**, right superior temporal, **right middle temporal gyrus**, **right lateral occipital cortex**, left and **right paracingulate gyrus**, left frontal pole, left and **right paracingulate gyrus**	**right temporal pole**, left and right subcallosal cortex, **right hippocampus and amygdala**, right and left cingulate gyrus, **right thalamus**
***Component 3*** ***Psychosis***	P-T1	left superior frontal gyrus, **left cingulate gyrus (anterior division)**, left lingual gyrus	**right occipital fusiform gyrus**, left lateral occipital cortex, **bilateral occipital pole**, right lingual gyrus, left cuneal cortex, right putamen, bilateral precentral gyrus
	P-T2	right frontal pole, **bilateral orbitofrontal cortex**,** bilateral amygdala**,** putamen**, left and **right thalamus**, **left** and right **frontal operculum**,** right paracingulate gyrus**, right superior parietal lobule, right middle frontal gyrus, **right supplementary motor cortex**, left lingual gyrus, right occipital pole	left orbitofrontal cortex, **left** and right **frontal pole**, **left superior frontal gyrus**, **left** and right **middle frontal gyrus**, right precuneus
	P-T3	right inferior temporal gyrus, **right temporal fusiform cortex**,** left temporal pole**,** left** and right **parahippocampal gyrus**,** left** and right **amygdala**,** bilateral insular cortices**, right middle temporal gyrus, **right supramarginal gyrus**,** right cingulate gyrus**, right middle frontal gyrus, **right superior frontal gyrus**,** bilateral lateral occipital cortex**,** right precuneus**	left and **right temporal fusiform cortex**,** right inferior**,** middle and superior temporal gyrus**, **right Heschl and supramarginal gyri**,** right angular gyrus**, left lingual gyrus, right lateral occipital cortex, **right postcentral gyrus**, **right parietal lobule**, **left middle frontal gyrus**, **bilateral superior frontal gyri**

**Fig. 4:**
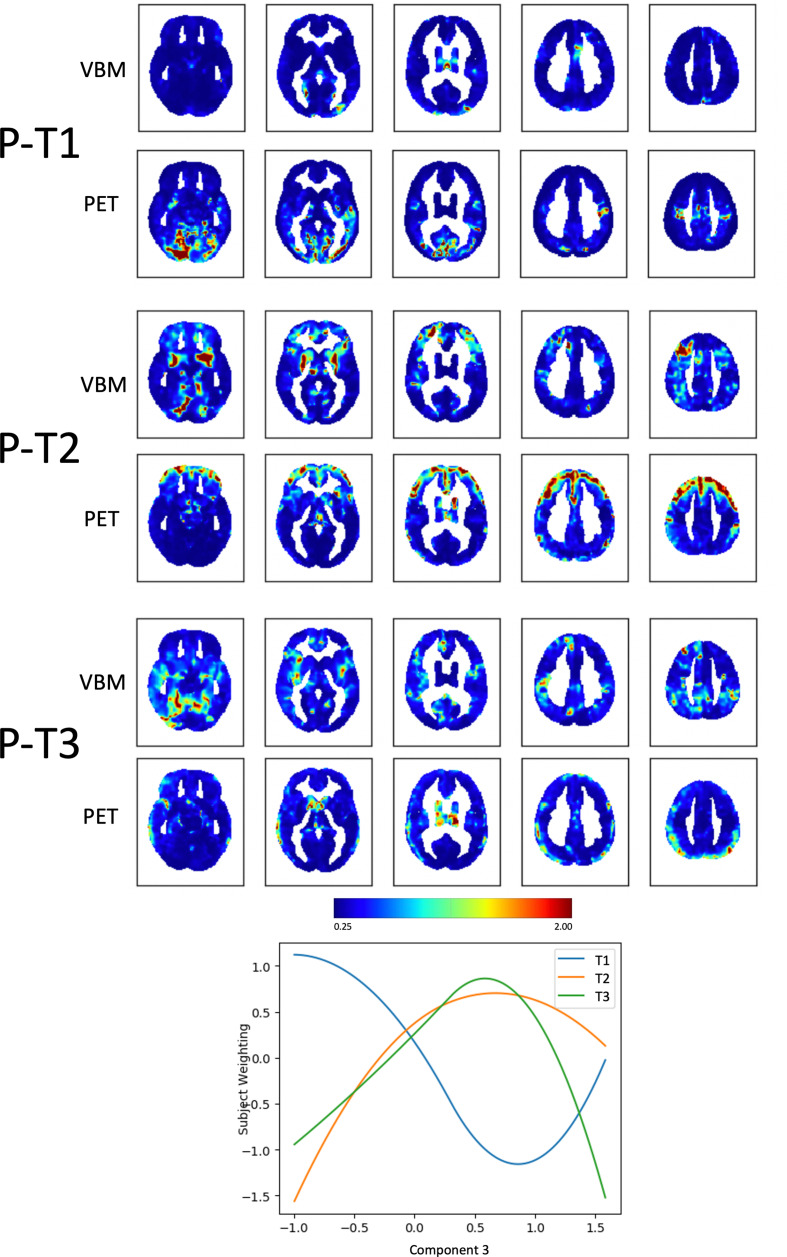
Fusion analysis on *Psychosis (P)* Spatial maps of clusters of voxels changing in tandem across the two modalities (VBM and PET) along the three trajectories (T) that “fit best” with the variability along Component 3, *Psychosis (P)*. The plot shows how the identified trajectories relate to Component 3. The color bar represents the likeliness for that voxel to belong to a given trajectory in terms of inverse and normalised squared Euclidean distance to each of the estimated centroid trajectories (varying from negligible [0 mm, blue], to average [0.6–0.8 mm, green-yellow], to high [> 1.2 mm, red])

Regions resulting from the multimodal fusion analysis. In bold, regions with the highest significant relationship with the behavioural components (i.e., those shown in red in Figs. 2, 3 and 4)

Component 2, *Disinhibition versus depression/mutism* (D), could be explained by 3 trajectories representing clusters of voxels changing in tandem across the two modalities (Supplementary Fig. 3B). Subject weights were plotted against Component 2 to visualize the extracted trajectories (Fig. 3; Table 2). The first trajectory (D-T1) captured brain voxels in which metabolism decreased with increasing values of depressive mood, stillness, and mutism: it highlighted larger clusters of voxels in the PET rather than in the VBM, mainly located in the left fronto-opercular region and in the sensory-motor cortex bilaterally. Another trajectory (D-T3) captured clusters of voxels in which volume and metabolism decreased with increasing disinhibition. These clusters were larger in the VBM than in the PET and were located for VBM only in the insular cortices bilaterally and right anterior cingulate, and for PET only in the right temporal pole.

Component 3, *Psychosis (P)*, could also be explained by 3 trajectories (Supplementary Fig. 3C). These trajectories represent clusters of voxels changing in the same way across the two imaging modalities in relation to the variability of Component 3. Subject weights were plotted against Component 3 to visualize the extracted trajectories (Fig. 4; Table 2). A first trajectory (P-T1) had almost the shape of the letter “U”, i.e. it captured voxels that had the lowest metabolism in the middle ranges of Component 3, and the highest metabolism in both the lower and upper ranges of the Component. In other words, this trajectory depicted clusters of voxels in which metabolism *was the highest* for high scores on hallucinations and delusions on Component 3, but also for low scores on the same Component. Clusters emerged mainly from PET and were in the occipital and auditory cortices. Another trajectory (P-T2) captured clusters in which volume and metabolism decreased for high values of hallucination and delusions. It captured clusters of increasing atrophy in the basal ganglia bilaterally and left lingual gyrus from the VBM and clusters of hypometabolism in the prefrontal cortex bilaterally in the PET. P-T3 captured clusters in the occipito-temporal gyrus, in the insula bilaterally and in the right cingulate cortex in the VBM, and in the basal ganglia (especially left thalamus and caudate) in the PET.

## Discussion

The aim of the present work was to improve our understanding of the structural and functional basis of the constellation of behavioural symptoms of FTD, by studying them with data-driven approaches, and relating them to two different imaging modalities (structural MRI and FDG-PET) in combination and non-linearly.

We found that the variability of behavioural and psychological symptoms in an FTD cohort was best captured by three components, which we labelled as (i) *Apathy*, (ii) *Disinhibition versus depression/mutism*, and (iii) *Psychosis*.

The fact that ratings for apathy and disinhibition from behavioural questionnaires (such as the FrSBe and the NPI) loaded on two different components contributes to the ongoing discussion on whether apathy and impulsivity represent opposite ends of a one-dimensional continuum or rather they tend to co-occur. Earlier clinical-anatomical studies aimed at capturing the variability of the constellation of behavioural and psychological symptoms in the FTD spectrum had identified two presentations with distinct neural correlates: one predominantly characterized by disinhibition and impulsivity, and the other predominantly characterized by apathy and inertia (Le Ber et al., 2006; Snowden et al., 2001; Zamboni et al., 2008). Whereas these early studies assumed that such “disinhibited” and “apathetic” profiles were the opposite ends of a behavioural continuum (i.e., a patient could have one *or* the other presentation), it has been now demonstrated that disinhibition and apathy usually co-occur in the same patient with FTD (Kok et al., 2021; Lansdall et al., 2017; Peters et al., 2006), and often coexist in cognitively healthy young individuals (Petitet et al., 2021). Our findings support the hypothesis that apathy and impulsivity may coexist to variable degrees but remain independent constructs with separate neuroanatomical correlates. They suggest that there may be patients who are both apathetic and disinhibited, as well as patients who are apathetic and depressed.

In addition to *Apathy*, our first Component also loaded ‘blunted affect’ and ‘decreased initiative’, possibly capturing, respectively, those that have been indicated as the *emotional* and *motivational* aspects of apathy (Ducharme et al., 2018). This component also loaded the FrSBe’s ‘executive dysfunction’ and the NBRS’s ‘poor planning’ and ‘conceptual disorganisation’. The association between apathy and executive dysfunction, which had also been shown in several previous studies (Eslinger et al., 2012; McPherson et al., 2002), suggests that this Component also captured what has been indicated as the *cognitive aspect* of apathy, pointing out the complexity of the “apathetic” phenotype in FTD (Ducharme et al., 2018).

The second Component, labelled as *Disinhibition versus depression/mutism*, was the only component that loaded specific behavioural disturbances on both its negative and positive ends. More precisely, it contrasted ‘disinhibition’ with ‘depressive mood’, highlighting aspects of disinhibition related to mania and abnormally elevated, expansive mood. But it also contrasted ‘disinhibition’ with ‘expressive deficit’ and ‘speech articulation defect’, highlighting aspects of disinhibition related to the prepotent verbal response and excessive garrulous chatter that FTD patients may present. Thus, we may assume that Component 2 captured several aspects of the multifaceted phenomenon associated with the broad term ‘disinhibition’, including those reflecting enhanced impulsivity or hyperactivation of the processes that generate the impulse, as well as those related to the loss of the knowledge of social rules or impairments in the suppression of prepotent responses and resistance to distractor interference (Magrath Guimet et al., 2021; Migliaccio et al., 2020). Importantly, this component did not change when we excluded patients that had started with language disturbances from the PCA, suggesting that it was not simply driven by their aphasia but rather captured behavioural variability across the different presenting phenotypes.

The third Component, labelled as *Psychosis*, remained stable and distinct even when increasing the number of extracted components in the PCA. This is consistent with findings in several previous studies (Aalten et al., 2008) and with the hypothesis that psychotic symptoms identify a specific phenotype in dementia (Ballard et al., 2020; Murray et al., 2014).

The second aim of the present study was to examine how the identified components of behavioural variability relate to changes in brain structure (MRI) *and* metabolism (FDG-PET). We preliminarily studied each modality separately with regression models exploring linear correlations: the unimodal VBM results were consistent with previous studies that had performed VBM correlational analyses of single behavioural questionnaires (Rosen et al., 2005; Sheelakumari et al., 2020; Zamboni et al., 2008). Interestingly, there were no regions of significant correlation between grey matter volume and *Psychosis* (Component 3). This is not surprising since few previous VBM studies had succeeded in the identification of significant linear correlations between psychosis and atrophy in patients with FTD, and their results did not survive corrections for multiple comparisons (Devenney et al., 2017; Sellami et al., 2018).

By using a newly developed fusion analysis we then studied, for the first time, how the identified components of behavioural variability relate to the two imaging modalities *in conjunction*, i.e., whether they are mainly associated with changes in structure (MRI), metabolism (FDG-PET), or both. In fact, it would be reasonable to think that some symptoms may mainly derive from alterations in the metabolism and not be associated with detectable atrophy, which takes longer to occur. Some other symptoms, instead, may be a direct consequence of the neurodegenerative process, which causes cell death and synapse loss, seen as focal grey matter atrophy. In addition, our multimodal decomposition technique allowed us to also uncover *nonlinear* relationships, as depicted by the trajectory plots often showing relationships that were flat for some portion and then changed or were even U-shaped, whereas previous studies had mainly searched for linear relationships.

The fusion analysis of MRI and PET data showed that voxels in which grey matter volume and metabolism decreased with increasing values of *Apathy* were mainly located in the anterior insula and anterior cingulate cortex, regions known to be part of the salience network (SN), and with hypometabolism in the right prefrontal cortex. The SN is specifically thought to be involved in detecting and processing salient information (Seeley et al., 2007). Another trajectory showed that increasing values on the A*pathy* component were associated with decreasing volume in the right cingulate and bilateral putamen, paired with largely decreased metabolism in the right prefrontal cortex. These two trajectories of multimodal covariation seem to capture what has been indicated as the motivational and cognitive components of apathy, respectively (Ducharme et al., 2018). Interestingly, in both trajectories a decreasing volume for bilateral subcortical structures was associated with hypometabolism of the right prefrontal cortex.

The fusion analysis on the *Disinhibition versus depression/mutism* component identified a trajectory with large clusters of hypometabolism, more than for atrophy, associated with increasing depression, mutism, and stillness in the left prefrontal cortex and in the sensory-motor cortex bilaterally. These regions have been associated, respectively, with language production, motor control, and depression (Davis et al., 2010; Ray et al., 2021). Another trajectory of the same Component captured instead clusters of atrophy, which was predominant for this trajectory, associated with increasing disinhibition, which was localised in the anterior insula bilaterally and right anterior cingulate. In addition, hypometabolism also involved the temporal poles. According to one functional interpretation of frontal-subcortical circuits (Tekin & Cummings, 2002), temporo-limbic structures are part of the orbitofrontal circuit, whose dysfunction is characterized by disinhibition syndromes including irritability, impulsivity, and undue familiarity. This has been interpreted both as *primarily frontal*, i.e., due to the loss of inhibition by the frontal monitoring system on the limbic system responsible for instinctual behaviors (Cummings, 1995), but also as *primarily subcortical*, i.e., due to the impaired risk perception mechanisms (Ghika, 2000).

Lastly, the fusion analysis on the *Psychosis* component mainly showed results from PET rather than VBM, suggesting that the symptoms described by Component 3 have greater functional rather than structural substrates. Among the trajectories associated with increasing scores of ‘psychosis’, one showed small clusters of atrophy in the basal ganglia (striatum) from the VBM and larger clusters of hypometabolism in the prefrontal cortex bilaterally from the PET. This component may capture the mesolimbic dopaminergic pathway, the dysfunction of which has been associated with positive symptoms in schizophrenia (McCutcheon et al., 2018, 2019). Another multimodal trajectory showed that increasing scores of psychosis are also associated with increasing metabolism in visual and auditory cortices, in line with the hypothesis that psychotic productive symptoms derive from aberrant overactive primary sensory areas (Alderson-Day et al., 2016; Zmigrod et al., 2016).

In summary, the fusion analyses indicated that some components of behavioural variability in FTD, such as *Apathy* and *Disinhibition versus depression/mutism*, were predominantly associated with changes in brain structure (atrophy). This suggests that apathy and disinhibition, like several other neurological symptoms, may occur as a consequence of brain damage/neuronal loss. The *Psychosis* component, instead, was predominantly associated with changes in brain metabolism (PET). This suggests that psychotic symptoms, like most productive symptoms, occur as a consequence of aberrant functioning of the brain. If confirmed by further studies, this observation may have important clinical implications. As an example, in the hypothetical clinical situation in which a patient with suspected bvFTD with persecutory delusions has a normal structural MRI, the clinician should not be put off by the lack of atrophy: in this case, FDG-PET is expected to be the meaningful imaging modality and should be prioritised. On the contrary, in the hypothetical clinical situation in which a patient with behavioural changes that are mainly characterised by apathy or disinhibition has a normal FDG-PET, the clinician should not be put off by the lack of hypometabolism: in this case, MRI is expected to be the meaningful imaging modality and should be prioritised.

This study has limitations. First, we lack a control group, therefore the identified brain-behavior correlations can only be interpreted as specific to FTD. Second, the study population was almost entirely Caucasian and non-Hispanic, limiting the generalization of our findings. Third, we only used observational questionnaires rather than experimental measures: this was in line with our aim to identify a framework that could be easily clinically interpreted. Lastly, we used a novel approach for decomposing multimodal neuroimaging data into distinct trajectories and associated spatial maps. Although it produced interpretable results consistent with the previous literature on the unimodal imaging correlates of behavioural disturbances, further validation of this method on a wide range of different datasets with different neurodegenerative populations would be useful.

## Conclusions

Our results suggest that there is a hierarchy in the way two imaging modalities (MRI and FDG-PET) relate to behavioural disturbances in FTD. Some behavioural disturbances appear to be predominantly associated with changes in brain structure/atrophy (measured by MRI). Others, such as *Psychosis*, with changes in brain function/metabolism (measured by FDG-PET). The clinical presentation may guide the choice of the neuroimaging investigation that should be prioritised in FTD patients.

## Electronic supplementary material

Below is the link to the electronic supplementary material.

Supplementary Material 1

## Data Availability

Anonymized data, including raw and analysed data, are available upon request to the first and senior authors.

## Code Availability

The code of the multimodal decomposition technique will be made available upon request to ZA and MJ.
