# Supplementary material for: Multimodal nonlinear correlates of behavioural symptoms in frontotemporal dementia
Source: Brain Imaging Behav. 2024 Sep 7;18(5):1226–38. doi: 10.1007/s11682-024-00913-7 (PMC11582133; doi:10.1007/s11682-024-00913-7)
Supplement: Supplementary file 1 — Supplementary Material 1 [file 11682_2024_913_MOESM1_ESM.docx]

**Supplemental methods**

**Multimodal decomposition technique**

The steps of the multimodal analysis, represented graphically in Supplementary Figure 1, are as follows. Initially we have *N* subjects, *K* modalities, where each modality has $M_{k}$ voxels. The following steps are applied for each variable of interest separately (in our case the behavioral components).

**Step 1.** Remove voxels that are not showing any significant change with respect to the variable of interest. We do this by binning the subjects into *B* bins (the value of *B* is discussed in the next step) based on the variable of interest and conducting several unpaired two-tailed t-tests to compare a given voxel's values in a bin with the voxel's values in all the other bins. If there were 10 bins, this means that for each voxel we would have to conduct (10^2^ - 10)/2 = 45 separate t-tests. We subsequently remove any voxels that do not show a significant change after Bonferroni correction for any of their t-tests, i.e. voxels that did not show a significant change across bins. This leaves a reduced dataset of size *N* subjects by $M_{k}^{\mathrm{Reduced}}$ voxels for modality *k*. Note in the flow chat $M_{k}^{\mathrm{Reduced}}$ is abbreviated to $M_{k}^{r}$. One benefit of this step is that hierarchical clustering initially creates a voxel-by-voxel distance matrix, and reducing the number of voxels would enable the distance matrix to fit into memory more easily. Another benefit is that the analysis will be less affected by noise from unimportant voxels.

**Step 2.** For each modality, bin each subject's data into the same *B* bins from step one and for every voxel average subject values within each bin. This leaves a bin-averaged dataset of size *B* bins by $M_{k}^{r}$ voxels for modality *k*. The binning reduces the dimensionality prior to clustering, reducing the computational burden and making the clustering more robust. Ideally the bins would be of equal width, have centres that are equally spaced within the range of the variable of interest and contain roughly equal numbers of subjects (although if the data does not allow it the method allows for different size bins to reach a compromise). Taking this into account, in this study we had 7 equally spaced bins for Apathy and Disinhibition versus depression/mutism, and 6 equally spaced bins for Psychosis. Binning reduces the computational burden and makes the clustering more robust since we go from clustering voxels in an *N*-dimensional space to a *B*-dimensional space.

**Step 3.** Concatenate the data across modalities to obtain a dataset of size *B* bins by $M^{Concatenated,Reduced}$ voxels. Note in the flow chat $M^{Concatenated,Reduced}$ is abbreviated to $M^{c,r}$. Note it is possible to also apply this method in a unimodal way by skipping this step and applying the rest of the steps for each modality separately. Only multimodal analysis using this method was done in this study how. Additionally, the original work of Arya et al. suggests that, prior to concatenation, to multiply all the values of one or more modalities by -1 to keep expected directions of change with respect to the variable of interest consistent if there is a strong prior belief for this. However, this was not needed for this study.

**Step 4.** Demean and variance normalise the values for each voxel. The bin-averaged values for each voxel represent a 'trajectory' of how that voxel's values change with respect to the variable of interest. The reason for demeaning and variance normalising is that we are interested in identifying trajectories that have different shapes irrespective of the scale or initial offset, and this is what allows voxels from different voxels and modalities to be clustered together if they show the same characteristics in their trajectories.

**Step 5.** Feed the dataset into ward hierarchical clustering, which minimises the variance of clusters being merged in a hierarchy, to obtain *L* clusters. The intuition is to merge voxels/sub-clusters that show most similar trajectories first. If a dataset contains clearly separable clusters, then the distance required to merge these real clusters should dramatically increase relative to each cluster's children. *L* now represents the number of trajectories/components that the data has been decomposed into and this should become visible on a dendrogram. Various automated methods for determining the correct number of clusters exist. However, real datasets are unlikely to contain clearly separable clusters, and so in this study we went with Arya et al.’s original approach of the visual inspection of the dendrograms as a way of choosing the most appropriate number of clusters. The number for *L* was typically 3 in this study, in favour of fewer components for easier interpretation. This results in *L* binary mask vectors, of length $M^{c,r}$ voxels.

**Step 6.** Calculate the subject weightings corresponding to each trajectory: the subject weights are derived from averaging the voxels values for a patient within the label mask, after ensuring the same normalization factors from step 4 are applied for consistency. A ‘trajectory’ refers to how voxel and/or component values change across the variable of interest. During the clustering step, the trajectory is with respect to bins as previously discussed, and so after clustering it is possible just take the ‘centroid’ trajectory, which would give a representative trajectory for that label. However, this would look sparse since the patients are binned prior to clustering. The subject weightings are derived by going back to the original data and averaging the voxels values for a patient within the label mask, after ensuring the same scaling factors from step 4 are applied for consistency, and normalising by the number of voxels in the binary mask. We now have a vector *N* subject weights for each *L* trajectory. In this study, the trajectory plots that we show are spline fits to the subject weights.

**Step 7.** Obtain continuous spatial maps corresponding to each trajectory. Step 5 gives binary label maps, and only for voxels that passed the initial pruning from Step 1. The aim of this step is to instead derive a value between 0 and 1 for all voxels, with this value being representative of the likelihood for that voxel to belong to a given trajectory, and constrain the values for a voxel to sum to 1 across all components. The whole-voxel data is binned and scaled as before, and the squared Euclidean distance to each of the *L* estimated centroid trajectories after Step 5 is calculated. This is inversed and normalised so that values sum to 1 across trajectories for a given voxel. This enables a more fine-grained and continuous spatial map to be obtained for easier interpretation of the results. The resulting vectors can be reshaped back into spatial maps for each modality.

As the methods stands, all trajectories for a given run of the method are valid: in this study, the most interesting ones were discussed in more detail.

**Supplementary Figure 1**

**
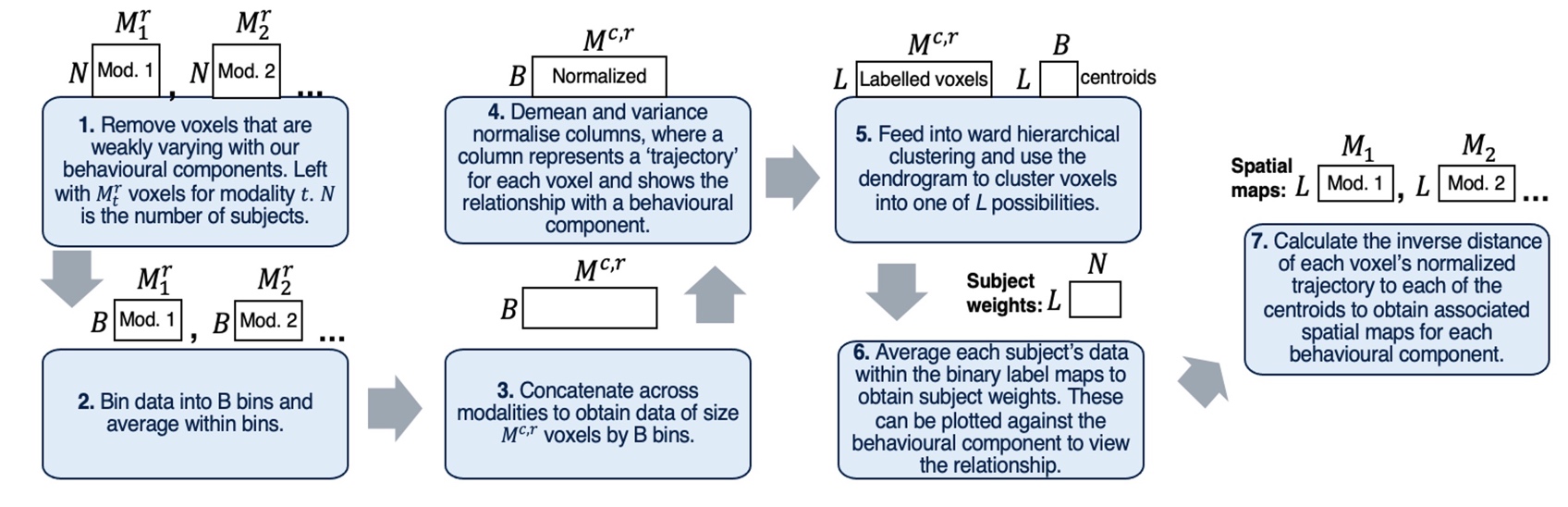
**

Flowchart representing the steps of the multimodal decomposition analysis.

**Supplementary Figure 2**


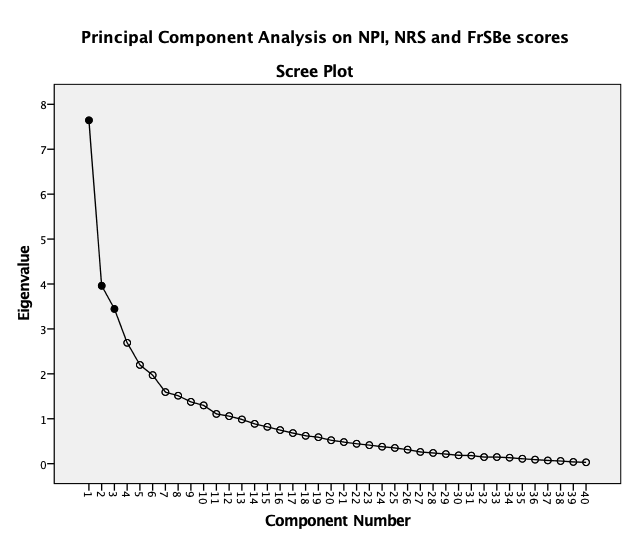


Scree plot of the eigenvalues obtained with the Principal Component Analysis.

FrSBe, Frontal Systems Behavior Scale; NBRS, Neurobehavioral Rating Scale; NPI, Neuropsychiatric Inventory.

**Supplementary Figure 3**

Dendrograms resulted from ward hierarchical clustering representing the number of trajectories that the data has been decomposed into.

A) Dendrogram obtained with hierarchical clustering for Component 1, *Apathy*


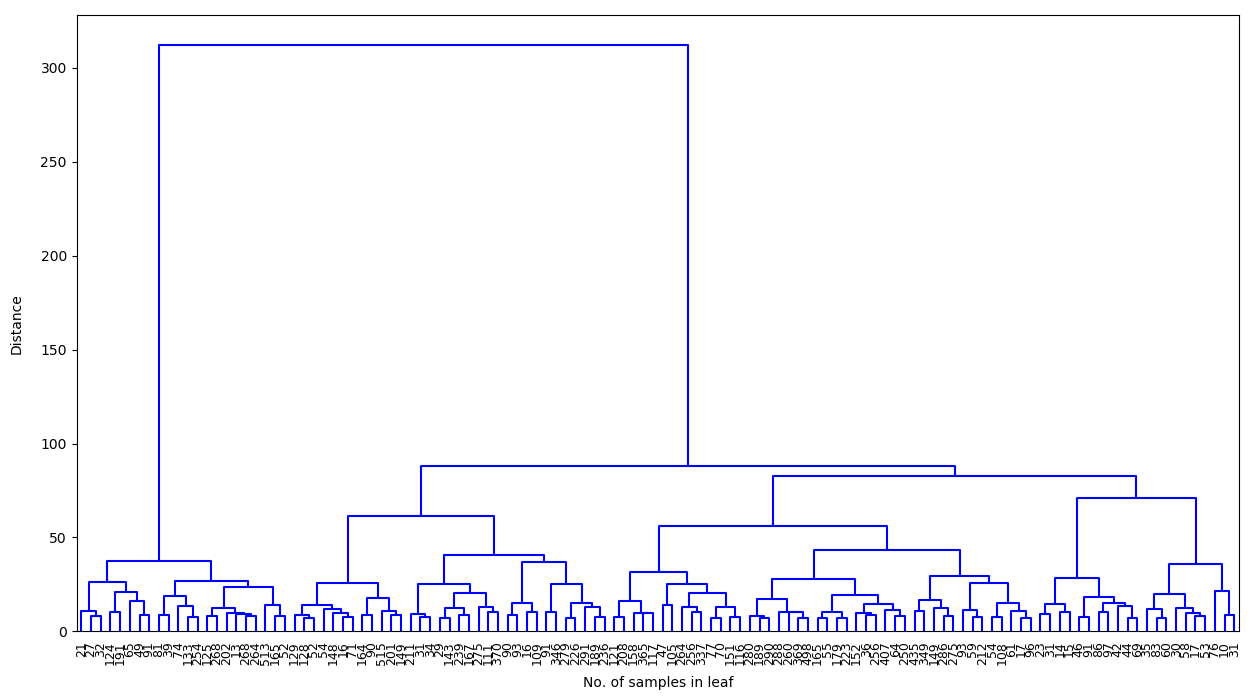


**B)** Dendrogram obtained with hierarchical clustering for Component 2, *Disinhibition versus mutism/depression*


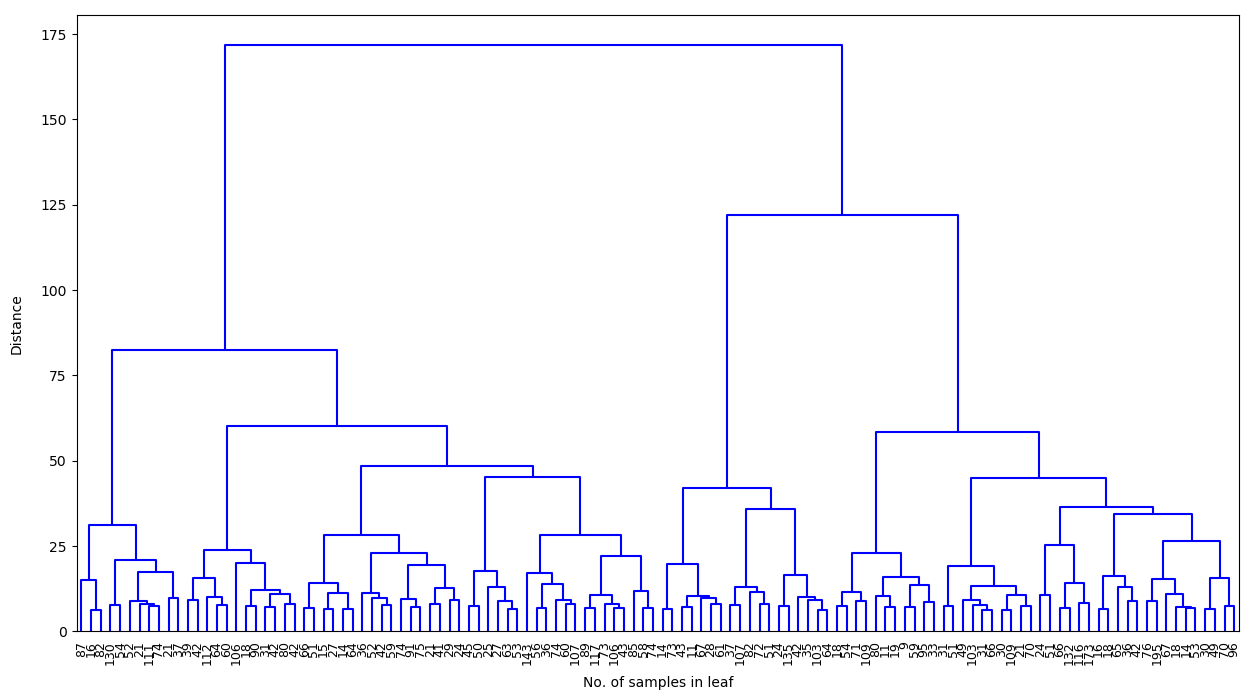


**C)** Dendrogram obtained with hierarchical clustering for Component 3, *Psychosis*


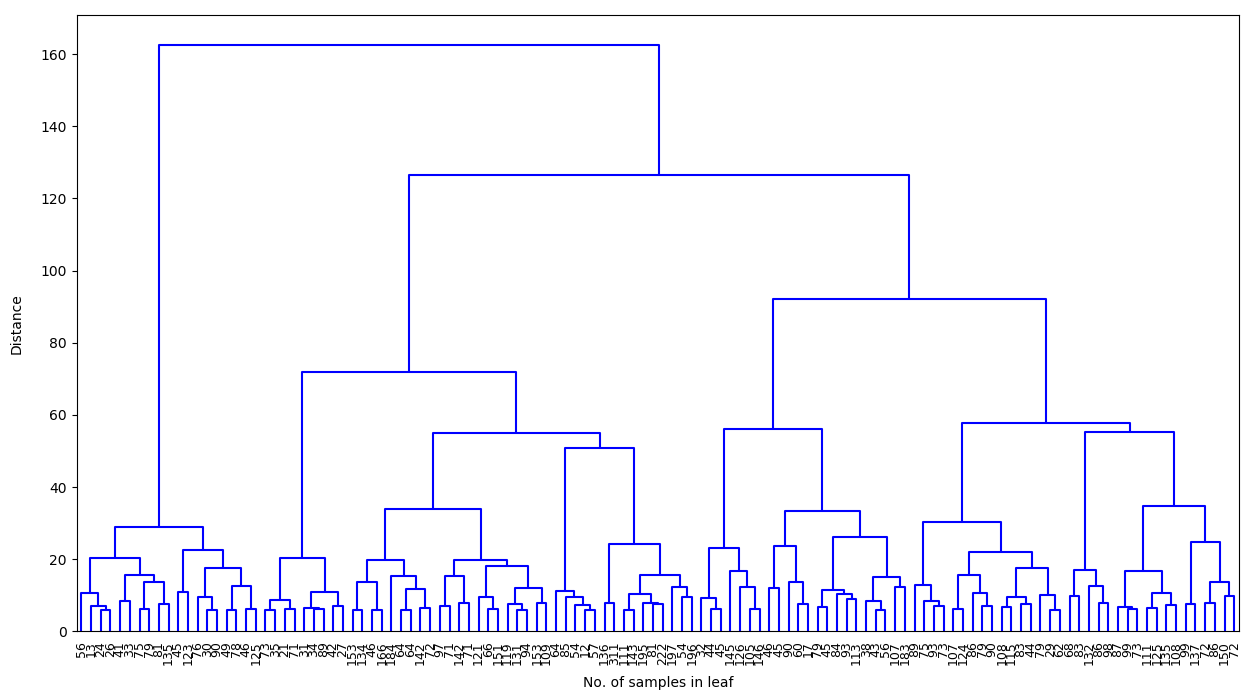


**Supplementary Table 1**

| **Input variable** | **Components** | | |
| --- | --- | --- | --- |
|  | Apathy | *Disinhibition versus mutism* | Psychosis |
| **Eigenvalue** | **7.644** | **3.961** | **3.445** |
| FrSBe (Caregiver) Apathy | **.695** | -.007 | -.032 |
| FrSBe (Caregiver) Disinhibition | .369 | **-.585** | -.057 |
| FrSBe (Caregiver) Executive Dysfunction | **.666** | -.211 | -.108 |
| NBRS Inattention/Reduced Alertness | **.762** | .101 | -.048 |
| NBRS Somatic Concern | -.042 | -.021 | .232 |
| NBRS Disorientation | .326 | .042 | -.253 |
| NBRS Anxiety | .012 | .012 | .513 |
| NBRS Expressive Deficit | .067 | **.611** | .018 |
| NBRS Emotional Withdrawal | **.741** | .222 | .160 |
| NBRS Conceptual Disorganization | **.636** | .112 | -.116 |
| NBRS Disinhibition | .316 | -.461 | .028 |
| NBRS Guilt Feelings | -.027 | .134 | -.001 |
| NBRS Memory Deficit | **.623** | .080 | -.072 |
| NBRS NBRS Agitation | **.536** | -.327 | .058 |
| NBRS Inaccurate Insight and Self Appraisal | .492 | -.362 | .160 |
| NBRS Depressive Mood | .173 | **.684** | -.146 |
| NBRS Hostility/Uncooperativeness | .314 | -.010 | .126 |
| NBRS Decreased Initiative, Motivation | **.779** | .174 | -.117 |
| NBRS Suspiciousness | .017 | .014 | **.669** |
| NBRS Fatigability | .356 | .172 | .231 |
| NBRS Hallucinatory Behavior | .009 | .070 | **.856** |
| NBRS Motor Retardation | .193 | .493 | -.154 |
| NBRS Unusual Thought Content | .051 | -.098 | **.715** |
| NBRS Blunted Affect | **.562** | .222 | .302 |
| NBRS Excitement | -.178 | -.447 | -.018 |
| NBRS Poor Planning | **.561** | .011 | .276 |
| NBRS Lability of Mood | .056 | -.013 | .031 |
| NBRS Tension | .203 | .105 | **.631** |
| NBRS Comprehension Deficit | **.557** | .258 | -.010 |
| NBRS Speech Articulation Defect | .124 | **.577** | -.126 |
| NPI Delusions | -.059 | -.222 | **.561** |
| NPI Hallucinations | .031 | .008 | **.700** |
| NPI Agitation | .425 | -.346 | .064 |
| NPI Depression/Dysphoria | .146 | .394 | -.029 |
| NPI Anxiety | .408 | -.275 | .199 |
| NPI Euphoria/Elation | .137 | -.446 | -.192 |
| NPI Apathy/Indifference | **.710** | -.203 | -.008 |
| NPI Disinihibition | .415 | **-.568** | -.114 |
| NPI Irritability/Lability | .299 | -.450 | .003 |
| NPI Aberrant Motor Behavior | .437 | -.250 | -.179 |

Rotated component matrix extracted from principal components analysis. FrSBe, Frontal Systems Behavior Scale; NBRS, Neurobehavioral Rating Scale; NPI, Neuropsychiatric Inventory
